# Comparing the Corrosion Resistance of 5083 Al and Al_2_O_3_3D/5083 Al Composite in a Chloride Environment

**DOI:** 10.3390/ma16010086

**Published:** 2022-12-22

**Authors:** Liang Yu, Chen Zhang, Yuan Liu, Yulong Yan, Pianpian Xu, Yanli Jiang, Xiuling Cao

**Affiliations:** 1 Key Laboratory of New Processing Technology for Nonferrous Metals & Materials, Guilin University of Technology, Guilin 541004, China; 2 Collaborative Innovation Center for Exploration of Nonferrous Metal Deposits and Efficient Utilization of Resources, Guilin University of Technology, Guilin 541004, China; 3 Guangxi Modern Industry College of Innovative Development in Nonferrous Metal Material, Guilin 541004, China; 4 Hebei Technology Innovation Center for Intelligent Development and Control of Underground Built Environment, Shijiazhuang 050031, China; 5 School of Urban Geology and Engineering, Hebei GEO University, Shijiazhuang 050031, China

**Keywords:** Al_2_O_3_3D/5083 Al, corrosion mechanism, electrochemistry, neutral salt spray, interface, interpenetrating structure

## Abstract

In this study, an Al_2_O_3_3D/5083 Al composite was fabricated by infiltrating a molten 5083 Al alloy into a three-dimensional alumina reticulated porosity ceramics skeleton preform (Al_2_O_3_3D) using a pressureless infiltration method. The corrosion resistance of 5083 Al alloy and Al_2_O_3_3D/5083 Al in NaCl solution were compared via electrochemical impedance spectroscopy (EIS), dynamic polarization potential (PDP), and neutral salt spray (NSS) tests. The microstructure of the two materials were investigated by 3D X-ray microscope and scanning electron microscopy aiming at understanding the corrosion mechanisms. Results show that an Al_2_O_3_3D/5083 Al composite consists of interpenetrating structure of 3D-continuous matrices of continuous networks 5083 Al alloy and Al_2_O_3_3D phase. A large area of strong interfaces of 5083 Al and Al_2_O_3_3D exist in the Al_2_O_3_3D/5083 Al composite. The corrosion development process can be divided into the initial period, the development period, and the stability period. Al_2_O_3_3D used as reinforcement in Al_2_O_3_3D/5083 Al composite improves the corrosion resistance of Al_2_O_3_3D/5083 Al composite via electrochemistry tests. Thus, the corrosion resistance of Al_2_O_3_3D/5083 Al is higher than that of 5083 Al alloy. The NSS test results indicate that the corrosion resistance of Al_2_O_3_3D/5083 Al was lower than that of 5083 Al alloy during the initial period, higher than that of 5083 Al alloy during the development period, and there was no obvious difference in corrosion resistance during the stability period. It is considered that the elements in 5083 Al alloy infiltrated into the Al_2_O_3_3D/5083 Al composite are segregated, and the uniform distribution of the segregated elements leads to galvanic corrosion during the corrosion initial period. The perfect combination of interfaces of Al_2_O_3_3D and the 5083 Al alloy matrix promotes excellent corrosion resistance during the stability period.

## 1. Introduction

The interpenetrating phase composites (IPCs) with percolating metallic and ceramic phases offer manifold benefits, such as a good combination of strength, toughness, and stiffness, very good thermal properties, excellent wear resistance, as well as the flexibility of microstructure and processing route selection, etc. [1]. The interconnectedness of the phases provides some promising benefits. Each phase contributes to the final composite’s properties, with the metal part increasing strength and fracture toughness compared to monolithic ceramics and the ceramic part increasing dimensional and mechanical stability at high temperatures compared to pure metal [2].

The fabrication of metal/ceramic IPCs typically involves two steps: (1) Processing of a reticulated porosity ceramic preform; (2) Infiltration of metallic melt in the pores of reticulated porosity ceramic preform to fabricate the IPC [3]. 

Al_2_O_3_3D/Al alloy IPC is one of metal/ceramic IPCs, which consists of 3D-continuous matrices of continuous networks Al alloy and Al_2_O_3_3D reticulated porosity ceramics phase [4]. Al_2_O_3_3D can inhibit the nucleation and growth of columnar crystals and reduce the area of exposed Al alloy matrix. Al_2_O_3_3D are used as reinforcement in Al_2_O_3_3D/Al alloy composite to achieve the high temperature mechanical and the wear resistance properties [5]. In the Al_2_O_3_3D/Al alloy IPCs, much of the driving force for investigating interpenetrating microstructures has been the toughening of Al_2_O_3_3D ceramic preforms by the addition of Al alloy metal phase [6]. Al_2_O_3_3D/Al alloy IPCs have a random, usually isotropic, spatial distribution of phases. Compared with particle-reinforced Al_2_O_3_P/Al, Al_2_O_3_3D/Al exhibits the advantages of high hardness, low density, high corrosion resistance, less corrosion spalling, and fewer defects. Due to their light weight quality, high mechanical properties, and excellent wear, Al_2_O_3_3D/Al alloy IPC is especially preferred for high temperature applications in many areas such as the automotive, space, and aviation industries [7,8,9].

Although Al alloys and aluminum metal matrix composites (AMCs) can improve the physical/mechanical properties, the presence of alloying elements and reinforcements can also increase the susceptibility to the more severe forms of localized corrosion: intergranular, exfoliation, and stress corrosion cracking [10]. Al_2_O_3_3D/Al alloy IPC corrosion often begins at the interface between the Al matrix and the composite reinforcements. Organic coatings with advantages of effectiveness and convenience have been widely applied to mitigate corrosion of AMCs, which can provide a robust physical barrier against the permeation of corrosive media [11]. The current protective methods to improve the corrosion resistance of the AMCs mainly include anodic oxidation, chemical conversion coating, surface facial mask layer, reinforcement surface coating and matrix alloying, etc. [12]. The heat treatment process, as well as the adjustment of reinforcement content and distribution, will also improve the corrosion resistance of AMCs [13].

The 5083 Al alloy is a high-magnesium alloy that exhibits good strength, corrosion resistance, good weldability, and machinability among non-heat treatable alloys [14]. In addition, 5083 Al alloy is widely used in maritime applications, automotive and aircraft weldments, and subway light rails for its excellent corrosion resistance [15]. Shuiqing Liu investigated the corrosion resistance of 5083 aluminum alloy after refining with nano-CeB6/Al, and found that nano-CeB6/Al inoculant showed a significant grain-refining effect on 5083 Al alloy, and the corrosion resistance of 5083 Al alloy was improved as well [16]. Roseline investigated the corrosion behaviour of heat treated Al metal matrix composites reinforced with fused zirconia alumina, and found the corrosion current density of the composites decreased with an increase in volume % of the heat-treated composite, comparatively more than the specimens that were not heat-treated [17].

Our previous research shows that, as a wear-resistant brake material, reticulated porosity SiC3D ceramic skeleton reinforced 6061 Al alloy metal composite (SiC3D/6061Al alloy IPCs) has excellent friction and wear performance, which can meet the requirements of high-speed train brake discs under emergency braking conditions [18] due to the SiC3D skeleton as a support to enhance the wear resistance of the material. However, obvious corrosion can be observed in the NSS corrosion experiment due to the weak interface bonding between SiC and Al [19]. It is worth improving corrosion resistance of IPC materials. 

In this work, reticulated porosity Al_2_O_3_3D ceramic skeleton and 5083 Al alloy were used to improve the corrosion resistance of IPCs materials. The Al_2_O_3_3D skeleton can reduce the area of Al matrix exposed to air and reduce the occurrence of corrosion reactions. Al_2_O_3_3D is tightly bonded to the Al matrix to reduce the defects of composites. In addition, the Al_2_O_3_ film generated by the oxidation of Al matrix adheres to the Al_2_O_3_3D skeleton. The research on corrosion mechanism of Al_2_O_3_3D/5083Al alloy IPC will promote industrialization of the high-speed train brake materials.

## 2. Materials and Methods

### 2.1. Experimental Materials

The reticulated porosity Al_2_O_3_3D ceramic skeleton (Al_2_O_3_3D) was prepared using a polymer replication technique. Polyurethane sponge (Shenzhen Green-tron Environmental Protection Filter Material Co., Ltd., Shenzhen, China) with 10 PPI porosity was used as a template to impregnate the Al_2_O_3_ slurry. Then, the excess Al_2_O_3_ slurry on the polyurethane sponge was removed. To improve the surface hanging slurry of the polyurethane sponge, the polyurethane sponge was dipped into 75 °C and 25 wt.% NaOH solution for 2 h to increase surface roughness. A round polyurethane sponge with dimensions of Φ = 500 mm and H = 100 mm was impregnated with Al_2_O_3_ covering slurry. The sponge was dried in a microwave oven for 15 min to obtain a green Al_2_O_3_ reticulated body with a good structure, cured at room temperature for 24 h, dried at 120 °C for 6 h. The Al_2_O_3_3D was produced in a graphite-resistance furnace (Jinzhou Santai Electric Furnace Factory, China) with argon gas as the sintering atmosphere. The sintering temperature was increased from 25 °C to 1600 °C with 2 °C/min, held at 1600 °C for 3 h, and cooled at room temperature to produce Al₂O₃3D.

**Figure 1:**
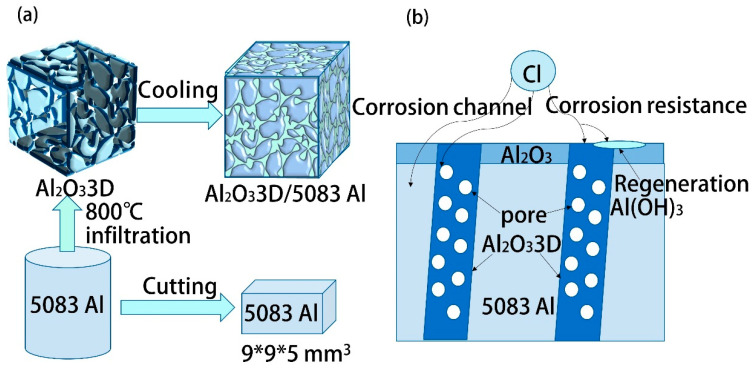
Preliminary preparation and corrosion direction: (**a**) preparation flow chart; (**b**) corrosion direction.

Figure 1 shows the preliminary preparation and corrosion direction. The pressureless infiltration method was used to create the Al_2_O_3_3D/5083 Al alloy composite. The volume ratio of 5083Al to Al₂O₃3D is about 8:2. 5083 Al alloy and Al_2_O_3_3D were put in two corundum crucibles, respectively, and both were heated to temperatures ranging from 25 °C to 800 °C. The liquid 5083 Al alloy in the crucible was manually agitated, and after being agitated, the aluminum solution was put back into the crucible which containing Al_2_O_3_3D. The Al_2_O_3_3D was gradually positioned on the aluminum solution and held there for 30 min. The aluminum solution was allowed to slowly infiltrate into the Al_2_O_3_3D and then cooled to obtain the Al_2_O_3_3D/5083 Al composite.

A metallographic cutting machine (Laizhou Weiyi Test Machinery Manufacturing Co., Ltd., Laizhou, China) was used to cut the Al_2_O_3_3D/5083 Al material into small squares that measured 9 × 9 × 5 mm^3^. A comparative study of corrosion performance was conducted with 5083 Al alloy. The composition of 5083 Al alloy is shown in Table 1. 

### 2.2. Characterization

Platinum sheet as the auxiliary electrode, and a sample as the working electrode, potentio-dynamic polarization (PDP) experiments and electrochemical impedance spectroscopy (EIS) were carried out on an electrochemical workstation (CHI 790E, Shang-hai Chenhua Instruments Co., Ltd., Shanghai, China). Hot-melt adhesive was used to bind copper wires (Jiangsu Jinzi Xuan Metal Technology Co., Ltd., Wuxi, China) to the back of each sample before it was put into a mold. The electrochemical test samples were made using proportional metallographic cold-mounting fluid (Shanghai Dental Materials Co., Ltd., Shanghai, China). The test sample’s working area was 0.81 cm^2^. The sample was polished with SiC sandpaper (Eagle) from 800 mesh to 1500 mesh, rinsed with deionized water, polished with 2.5 purpose metallographic polish, rinsed with deionized water, wiped with alcohol, and dried with a hair dryer in cold air.

Tests for open circuit potential (OCP), PDP, and EIS were performed on a sample of a polished surface. For the OCP testing and PDP investigations, the test samples were placed in a glass cell with 3.5 wt.% NaCl solution (Dongguan Xunye Chemical Reagent Co., Ltd., Dongguan, China) at room temperature for 6 min. The OCP testing hold was 1000 s with a starting potential of –1.3 V and a scan direction from cathode to anode, with a scanning interval of roughly 600 mV in relation to the self-corrosive potential, the scanning speed from cathode to anode direction was 0.25 mv/s. Samples of polished surface were measured at OCP stabilization by using a 10 mV perturbed potential sine wave for EIS, and samples were measured within the frequency range of 10^−2^–10^6^ Hz. Samples of polished surface were observed using a high-resolution 3D X-ray Microscope (Zeiss Xradia 510 Versa, Carl Zeiss) for the microstructure characterization of the samples.

A laboratory-prepared Keller solution (95 mL deionized water + 2.5 mL HNO_3_ + 1.5 mL HCl + 1.0 mL HF) was used to etch the specimens for the intergranular corrosion test. The samples were etched with Keller’s solution for 10–20 s, rinsed with deionized water, and wiped with alcohol. Sample tissues were observed using an inverted metallographic microscope (MS600 Hangzhou Jingke Testing Instruments Co., Ltd., Hangzhou, China). A neutral salt spray (NSS) corrosion test was performed using a fully automatic salt spray tester (ZK-60K, Dongguan Zhenke Testing Equipment Co., Ltd., Dongguan, China). The experiments were performed with 5% mass fraction of corrosive liquid and neutral NaCl solution with pH 6.5–7.2. The experimental times were 24, 72, 144, 240 and 360 h at (35 ± 1) °C. The experimental samples were observed using Zeiss GeminiSEM 300 field-emission scanning electron microscope (Oberkochen, Germany) equipped with an energy-dispersive X-ray spectrometer (Oberkochen, Germany) for the microstructure characterization of the samples and corrosion products.

## 3. Results

### 3.1. Sample of Polished Surface of the Two Materials

**Figure 2:**
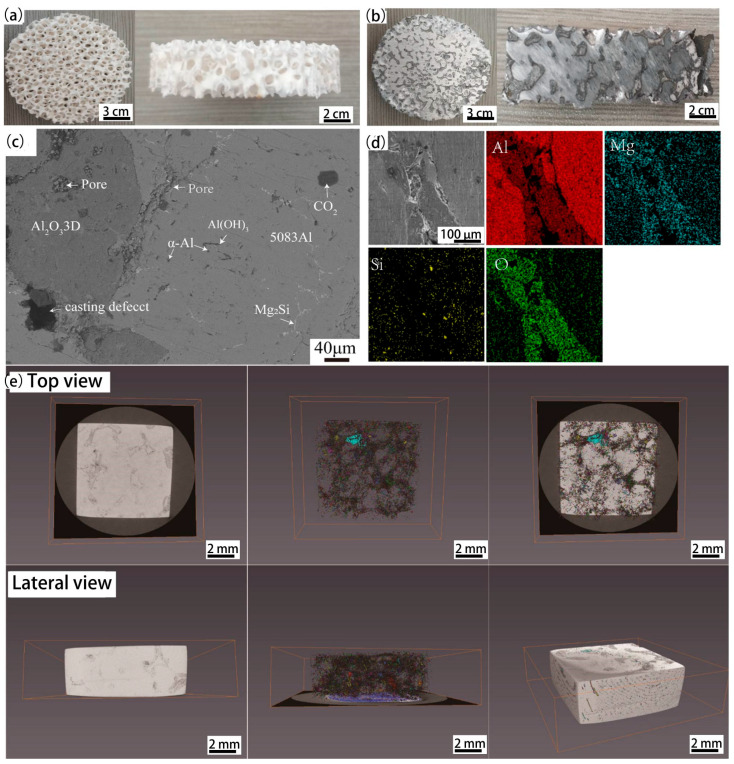
(**a**) Al_2_O_3_3D; (**b**) sampling point of Al_2_O_3_3D/5083Al; (**c**) SEM (Scanning Electron Microscope) of the Al_2_O_3_3D/5083Al; (**d**) EDS of the Al_2_O_3_3D/5083Al; (**e**) 3D XRM (High resolution 3D X-ray Microscope) of Al_2_O_3_3D/5083Al.

Figure 2a shows the burned round disks shaped Al_2_O_3_3D. The overall skeleton is white, round, and exhibits certain hardness and strength. The Vickers hardness tester measures compressive strength at 3.9 MPa. The flexural strength of sintered Al_2_O_3_ foams was determined from three-point bending. The loaded surfaces were covered with a thin sponge layer to obtain uniform load distribution throughout the faces. In all mechanical determinations, results were based on an average of five samples. The measured flexural resistance was 2.7 MPa. The pores of Al_2_O_3_3D are approximate round holes, and measured at 2–3 mm.

Figure 2b shows the prepared Al_2_O_3_3D/5083 Al composite. The prepared sample by a pressureless infiltration method shows typical IPC structural characteristics and exhibits a light silver metallic luster. The sample is compact in structure, without obvious pores on the outer surface, which implies it has high strength and tightness. 

Figure 2c shows the scanning electron microscopy (SEM) image of the Al_2_O_3_3D/5083 Al composite without corrosion. The brighter part is the 5083 Al matrix, while the darker part is the Al_2_O_3_3D. The second phase was observed in the 5083 Al matrix. The round dot is α-Al_2_O_3_, and the thin strip is Al_6_ (iron, manganese), Mg_2_Si. Because there is a large amount of Al_2_O_3_ powder in the green Al_2_O_3_ reticulated body, it is difficult to form a sintering neck during the sintering process. Consequently, Al_2_O_3_3D is not completely dense, leaving defects such as pores. In addition, in the process of molten 5083 Al liquid infiltrating into the Al_2_O_3_3D, the thermal stress of Al_2_O_3_3D is transferred and released in the direction of the Al_2_O_3_3D, causing damage to the Al_2_O_3_3D. The Al_2_O_3_3D has cracks and dark pits on the surface and inside, exerting a negative effect on the performance of the Al_2_O_3_3D/5083 Al composite [20].

Figure 2d shows the energy-dispersive X-ray spectroscopy (EDS) diagram of the uncorroded Al_2_O_3_3D/5083 Al composite. No delamination occurred between the 5083 Al matrix and the Al_2_O_3_3D, and the Mg appeared enriched in the Al_2_O_3_3D. During the preparation, the molten 5083 Al liquid released energy when it cooled down. The diffusion ability of the Mg in the molten 5083 Al liquid was considerably enhanced, and it entered through the Al_2_O_3_3D. The Al_2_O_3_ particles in the Al_2_O_3_3D exhibited an adsorption effect, showing the enrichment of the Mg in the Al_2_O_3_3D. During the cooling process of the 5083 Al liquid, the second phase precipitated. The EDS indicates a point-like enrichment of the Si, which exerts minimal effect on the performance of the Al_2_O_3_3D/5083 Al. The O demonstrated enrichment in the Al_2_O_3_3D [21].

Figure 2e shows the test diagram of the 3D X-ray microscopy (XRM) of uncorroded Al_2_O_3_3D/5083 Al. The pores are concentrated at the Al_2_O_3_3D. A few pores were observed on the surface of the Al matrix. .A large area of strong interfaces of 5083 Al and Al_2_O_3_3D was observed in the Al_2_O_3_3D/5083 Al composite. No evident delamination is found between the two phases.

**Figure 3:**
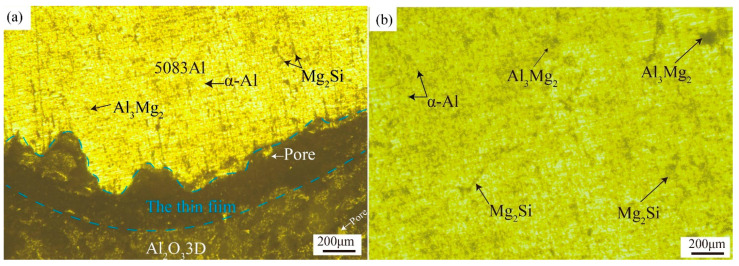
Optical micrograph (OM) image of two materials: (**a**) Al_2_O_3_3D/5083 Al; (**b**) 5083 Al.

Figure 3a shows the optical microscopy (OM) image of the Al_2_O_3_3D/5083 Al composite. The eroded out metallographic phase by the etching solution can be seen between the Al_2_O_3_3D and the 5083 Al matrix. The boundary has a thicker layer because, when the material is compounded, the aluminum liquid touches the Al_2_O_3_3D skeleton, resulting in faster cooling of the parts in contact. The aluminum liquid will preferentially solidify at the Al_2_O_3_3D skeleton, resulting in the uneven local solidification of the aluminum liquid. The Al_2_O_3_3D skeleton will have more solidified parts. During the preparation of Al_2_O_3_3D/5083 Al composites, second phases were precipitated, mostly Al_3_Mg_2_, α-Al, and Mg_2_Si [22].

Figure 3b shows the optical micrograph of the 5083 Al alloy. The Al also precipitates Al_3_Mg_2_, α-Al, and Mg_2_Si during solidification. The Al_2_O_3_3D in the composite can inhibit interfacial reaction, exerting a positive effect on material properties [23].

### 3.2. Polarization Curve

**Figure 4:**
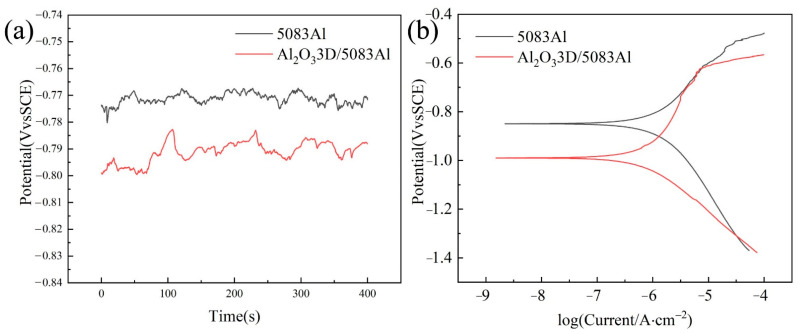
(**a**) Open circuit potential (OCP) curve; (**b**) potentio-dynamic polarization (PDP) curve.

The electrochemical corrosion of the Al_2_O_3_3D/5083 Al composite includes interfacial and intergranular corrosion. The OCP and PDP of the Al_2_O_3_3D/5083 Al composite and 5083 Al are depicted in Figure 4a,b. The OCP, corrosion potential (E_corr_), and corrosion current density (I_corr_) values obtained from the curves in Figure 4 are given in Table 2. The OCP voltage of the Al_2_O_3_3D/5083 Al composite is more negative than that of 5083 Al due to certain defects in the sample preparation.

In the polarization curve, the E_corr_ value of the Al_2_O_3_3D/5083 Al composite is smaller than that of 5083 Al, while its I_corr_ value is smaller than that of 5083 Al, indicating that the corrosion tendency of the Al_2_O_3_3D/5083 Al composite is higher than that of 5083 Al. The corrosion current is the determining factor of the corrosion resistance of the materials. The I_corr_ value of the Al_2_O_3_3D/5083 Al composite is smaller than that of 5083 Al, indicating that the corrosion resistance of the Al_2_O_3_3D/5083 Al composite is better than that of 5083 Al.

**Figure 5:**
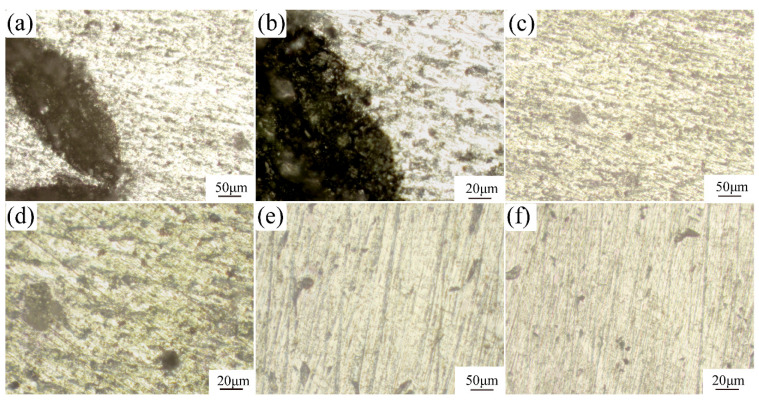
Optical micrographs of two materials after Tafel corrosion: (**a**–**d**) Al_2_O_3_3D/5083Al; (**e**,**f**) 5083 Al.

Figure 5 shows the optical micrographs after electrochemical corrosion. More Mg_2_Si particles precipitated in the Al_2_O_3_3D/5083 Al composite in Figure 5d than in the 5083 Al alloy in Figure 5f. The 5083 Al matrix in the Al_2_O_3_3D/5083 Al composite exhibits a higher tendency to corrode and is less sensitive to early pitting microporous nucleation due to the small potential difference between Mg_2_Si particles and the 5083 Al matrix. 

During the corrosion process, Mg_2_Si particles hindered the continuity of the matrix and inhibited corrosion [24]. The precipitation of the second phase reduces the corrosion sensitivity of the material, inhibits corrosion tendency, and reduces corrosion current density. Notably, the Al_2_O_3_3D/5083 Al composite not only exhibits the advantages of large interfacial composite, less interfacial concentration, small specific surface area, anticorrosive, and antioxidant IPC structure, but the Al_2_O_3_3D also simultaneously enhances the corrosion resistance of the Al_2_O_3_3D/5083 Al composite. In the preparation of the Al_2_O_3_3D/5083 Al composite, the Al_2_O_3_3D demonstrates strongly bonding properties with the 5083 Al to reduce the generation of voids. When the 5083 Al matrix in the Al_2_O_3_3D/5083 Al composite was oxidized, the Al_2_O_3_ film generated by oxidation combined with Al_2_O_3_3D to fill the voids of the composite, making the IPC structure denser.

### 3.3. EIS of Polished Surface Materials

**Figure 6:**
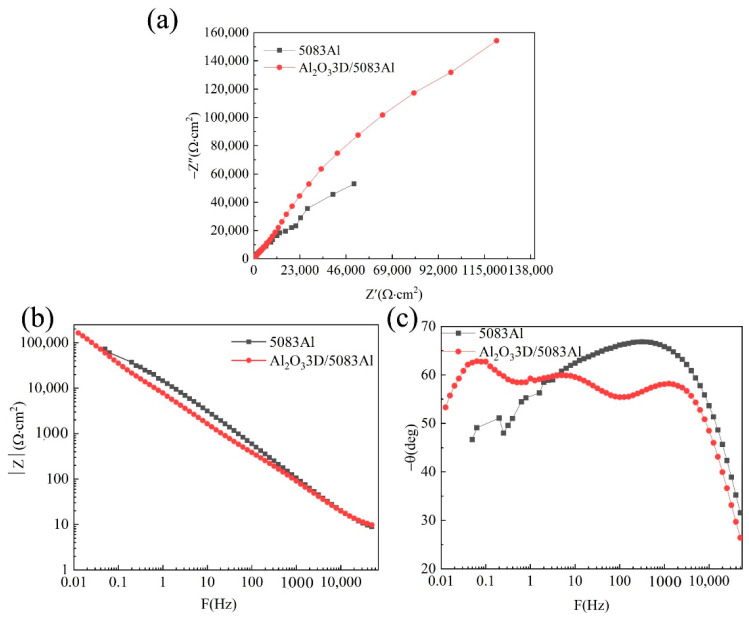
Uncorroded electrochemical alternating current impedance spectroscopy (EIS): (**a**) Nyquist diagram; (**b**) Bode diagram (|Z|-F); (**c**) Bode diagram (−θ-F).

Figure 6 shows the EIS plots of Al_2_O_3_3D/5083 Al and 5083 Al in the absence of salt spray corrosion. The Nyquist plots of two materials are capacitive reactance plots in Figure 6a. The impedance spectra are capacitive reactance arcs in the high-frequency region, reflecting the electrochemical reaction of corrosion on the electrode surface. The two materials show a similar EIS curve at high frequencies ranging from 1.0 × 10^5^–5.0 × 10^5^ Hz, and Al_2_O_3_3D/5083 Al is considerably larger than 5083 Al at low frequencies ranging from 0.01–10 Hz, indicating greater resistance and corrosion resistance of Al_2_O_3_3D/5083 Al [25]. The Nyquist plot in Figure 6a is a semicircle. Therefore, the control step of the electrode process is determined by the electrochemical reaction step (charge transfer process). The impedance caused by the diffusion process can be disregarded. Figure 6b,c show that the Bode plot has two time constants. The EIS results of Al_2_O_3_3D/5083 Al and 5083 Al are consistent with the PDP results presented in Figure 4.

### 3.4. Corrosion Morphology Analysis

**Figure 7:**
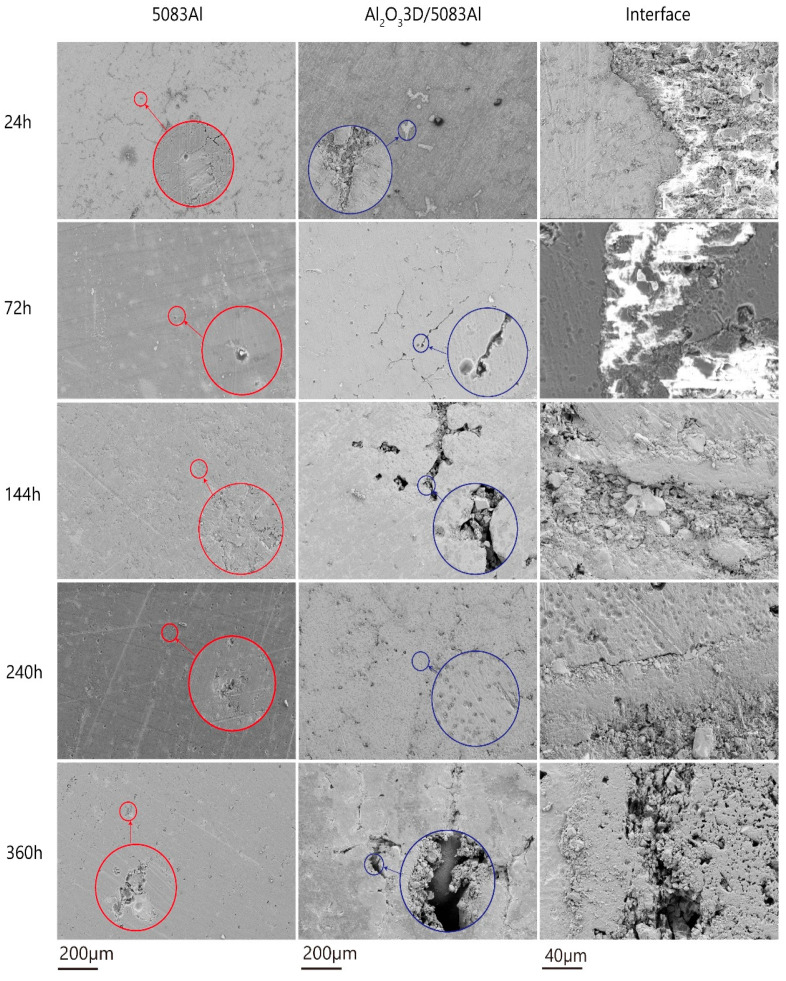
Corrosion morphology of Al_2_O_3_3D/5083Al and 5083Al under different times after NSS (neutral salt spray) corrosion.

The NSS tests show that the corrosion process of Al_2_O_3_3D/5083 Al and 5083 Al consists of pitting, intergranular corrosion, and spalling corrosion [26]. The surface morphology of the NSS-corroded Al_2_O_3_3D/5083 Al and 5083 Al specimens is shown in Figure 7, the red and blue circles in the figure are a partial enlargement of the original Figure 7. To observe the microstructure of the 5083 Al alloy and the degree of corrosion of 5083 Al matrix in an Al_2_O_3_3D/5083 Al composite, the metal phases of both materials were amplified and processed.

After 24 h, pitting appeared in 5083 Al. The size of pitting was about 0.5 μm, and the overall structure was well maintained. During the initial period, Al_2_O_3_3D/5083 Al has fine corrosion pits in the Al matrix. These pits defects were formed in the process of pressureless infiltration of Al_2_O_3_3D/5083 Al. At the bond of the interface, no evident corrosion pits and no evident damage of Al_2_O_3_3D are found [27].

After 72 h, a gradual increase in the pitting of 5083 Al was observed. During the initial period, pitting occurred around the precipitates, which was driven by the galvanic coupling effect. The pitting rapidly extended horizontally with shallow circular structures caused by the deposition around corrosion pits. Al_2_O_3_3D/5083 Al exhibited an increase in corrosion pits in the Al matrix, the oxide film on the surface was destroyed, no significant corrosion change occurred in the aluminum matrix at the boundary, and Al_2_O_3_3D presented corrosion pits.

After 144 h, 5083 Al pitting developed substantially, small pieces of spalling corrosion appeared, and the Al_2_O_3_ film broke down. During the development period, Al_2_O_3_3D/5083 Al demonstrated substantial development of corrosion pits in the Al matrix. The substrate Al was exposed, pitting deepened, with corrosion and evident reaction on a small area. No evident corrosion occurred on a large area, with pitting in the Al matrix at the interface and spalling of Al_2_O_3_ particles in Al_2_O_3_3D. 

After 240 h, 5083 Al spalling corrosion increased, accompanied by the development of pitting and cracking. During the development period, the structure of 5083 Al was not significantly damaged. The generation of Al_2_O_3_ appeared on the surface, and the self-healing of Al began to occur. Al_2_O_3_3D/5083 Al presented a large area of pitting in the Al matrix, accompanied by the deepening of local corrosion pits. The Al_2_O_3_ film on the surface was destroyed, and a large area of pits were observed at the interface. Corrosion developed downward along the pores of Al_2_O_3_3D.

After 360 h, 5083 Al spalling corrosion was enhanced, and large corrosion pits appeared in the spalled Al matrix and developed downward in depth. During the stability period, Al_2_O_3_3D/5083 Al corrosion pit depth was elevated in the Al matrix. Corrosion pits became larger, and pitting corrosion appeared on the interface between the Al_2_O_3_3D and 5083 Al two phases. Depth development of pitting corrosion occurred at the Al matrix, and the surface of Al_2_O_3_3D located at the interface was destroyed. Deepening and expansion of corrosion pits were demonstrated on Al_2_O_3_3D.

Therefore, 5083 Al present pitting after corrosion testing, milder than a composite metal matrix surface. Al_2_O_3_3D/5083 Al has a part of the corrosion enrichment of the Al matrix, and evident damage was observed. However, it exerted a minimal effect on the overall corrosion resistance, and IPC structure before and after corrosion is maintained well.

**Figure 8:**
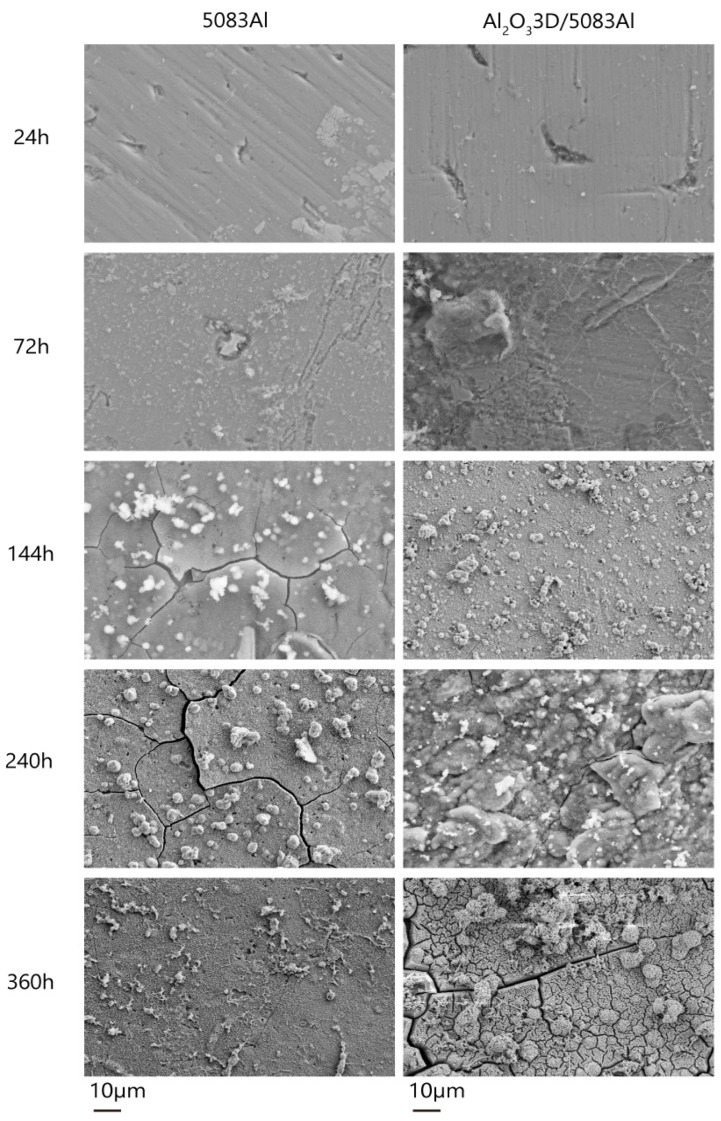
Corrosion product morphology under different times after NSS corrosion.

Figure 8 shows the morphology of the corrosion products of NSS-corroded Al_2_O_3_3D/5083 Al and 5083 Al. 

After 24 h, 5083 Al showed fine pitting with more pitting divisions accompanied by small pieces of Al (OH)_3_ adsorbed onto the surface. During the initial period, Al_2_O_3_3D/5083 Al showed pitting that was larger compared with that of 5083 Al but less in number [28]. The substrate Al was maintained well. 

After 72 h, 5083 Al showed deepening of pitting, and visible Al (OH)_3_ was generated on the corrosion pits. During the initial period, Pitting Al (OH)_3_ appeared around the corrosion pits. Al_2_O_3_3D/5083 Al exhibited partial deepening of pitting pits. Large pieces of Al (OH)_3_ appeared around the pitting pits, and the IPC structure was maintained well. 

After 144 h, 5083 Al showed corrosion cracks. Corrosion led to the formation of the second phase, where a rupture of the oxide film occurred. During the development period, Al exuded from the crack and was subjected to oxidation to generate pitting Al_2_O_3_. A small amount of Al (OH)_3_ enriched at the crack played a role in repairing the crack, and Al_2_O_3_3D/5083 Al showed a small area of pitting. Metal elements exuded at the pitting pits on the substrate Al, and a small amount of Al (OH)_3_ agglomerated at the pitting pits, increasing roughness of the surface. 

After 240 h, the pitting deepened on the surface of 5083 Al, causing a large expansion of cracks. During the development period, the passivation film was destroyed. The surface of Al_2_O_3_3D/5083 Al showed a deepening of pitting. A large number of elements exuded from the Al matrix and Al_2_O_3_3D. The Al element was oxidized on the surface, forming a new Al_2_O_3_ film with enhanced corrosion resistance. 

After 360 h, the surface of 5083 Al formed a porous oxide film layer. During the stability period, small agglomerates of Al (OH)_3_ appeared on the Al_2_O_3_ film of Al_2_O_3_3D/5083 Al. Meanwhile, repaired cracks reappeared. Spherical oxide particles were distributed around for the self-repair of the oxide film. 

**Figure 9:**
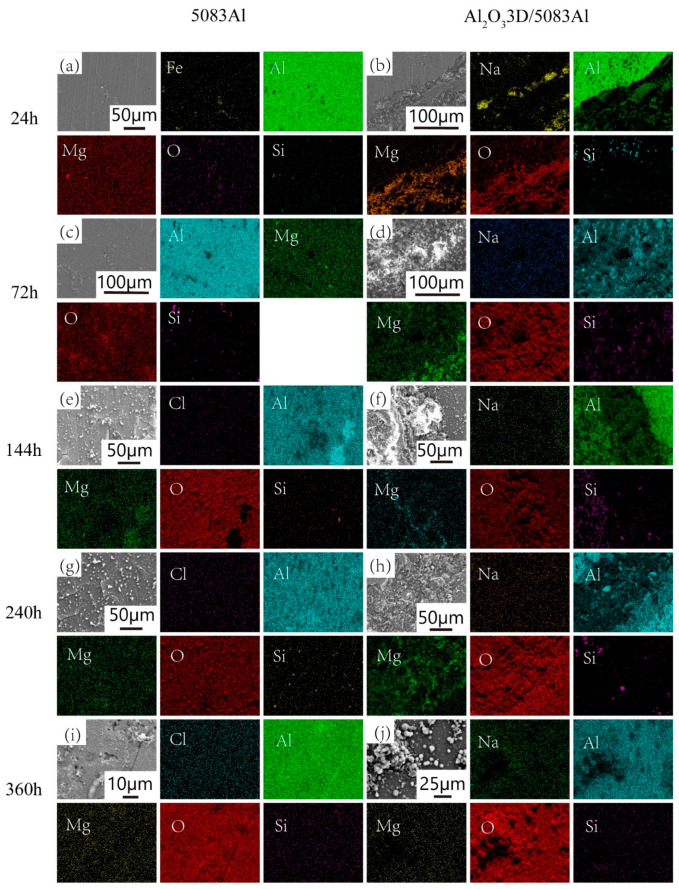
Energy dispersive X-ray spectrometer (EDS) of corrosion products with different times after NSS corrosion: (**a,c**,**e**,**g**,**i**) 5083Al; (**b**,**d**,**f**,**h**,**j**) Al_2_O_3_3D/5083Al.

Figure 9 shows the EDS of corrosion products at different times after NSS corrosion. The scale bar used for the elements is the same as that used for the corresponding microstructure. 

After 24 h, the EDS of 5083 Al showed no significant changes on the surface. During the initial period, Al and Fe atoms diffused out of the pitting and were oxidized. The EDS of Al_2_O_3_3D/5083 Al showed that the Mg was enriched toward the Al_2_O_3_3D during corrosion. 

After 72 h, a small amount of Si precipitated on the surface of the Al matrix in a punctuated division. During the initial period, an enrichment of Na occurred at the interface, and an aggregation of elements on the skeleton of Al_2_O_3_3D was observed. The Na on the surface of Al originated from the atmosphere, and Na was involved in the generation of corrosion products NaAlCO_3_(OH)_2_. The content of NaAlCO_3_(OH)_2_ decreased as the depth increased following an exponential power function.

After 144 h, the EDS of 5083 Al indicated that a small amount of Cl^-^ was uniformly divided on the surface. During the development period, Al atoms were gathered at the corrosion pits, and the oxide film was broken. Spalling corrosion exposed the aluminum substrate to air. The EDS showed that a large number of Mg atoms were precipitated at the corrosion pits. Corrosion was relatively fast, and Mg atoms acting as the anode were oxidized. The corrosion of the 5083 Al began as intergranular corrosion and galvanic corrosion. Si atoms were also gathered at the corrosion pits in the form of spots, with the active state of the damaged Al_2_O_3_ film acting as the anode and the passive state of the undamaged film acting as the cathode, constituting an activation–passivation cell, wherein the redox reaction caused the metal to dissolve in the pore. The pore was maintained as electrically neutral, with Cl^−^ migrating into the pore. The Al atoms were activated by the action of Cl^−^, forming a pore activation (internal)–passivation (external) corrosion cell with increased migration of Cl^−^. The EDS of Al_2_O_3_3D/5083 Al showed a small amount of uniform precipitation of Na atoms. Corrosion products of Al gathered on the Al_2_O_3_3D. Corrosion products of Mg atoms were distributed on both sides of the interface and enriched in the two-phase interface. O atoms were found on the surface of the uniform division, indicating that the oxide film structure was maintained well. Corrosion products of Si atoms were distributed on both sides of the interface. The performance of the division state was a small amount of aggregation at the interface, and a small amount of corrosion products on the surface of the Al matrix. The second phase generation was enriched at the interface, enhancing the corrosion resistance of the interface.

After 240 h, the EDS of 5083 Al presented a small amount of Cl^-^ that was uniformly distributed on the surface. During the development period, Al_2_O_3_ was generated again, with no vacancy or agglomeration of Al and Mg divisions, indicating that corrosion pits were covered, and corrosion resistance was enhanced. A small amount of Si was distributed on the surface, showing a dotted division. The precipitated second phase mixed with oxidation-generated Al_2_O_3_ encapsulated the metal surface. Corrosion products of Mg precipitated on the Al_2_O_3_3D. O divisions indicated that the Al_2_O_3_3D was bonded well with the interface of 5083 Al, and the oxide film was not damaged. Si divisions were concentrated at the interface.

After 360 h, the EDS of 5083 Al presented an increase in brightness of the residual Cl^-^ with an increase in corrosion time and an increase in the residual amount of Cl^-^. During the stability period, a small amount of Mg gathered around the corrosion pits, and O indicated the destruction of the oxide film. The corrosion products of Si decreased, and the secondary phase was consumed. The corrosion map of Al_2_O_3_3D/5083 Al showed Al (OH)_3_ encapsulation at the corrosion pits. O were distributed to agglomerate at the corrosion pits and covered the corrosion pits.

### 3.5. EIS of Corrosion Product

**Figure 10:**
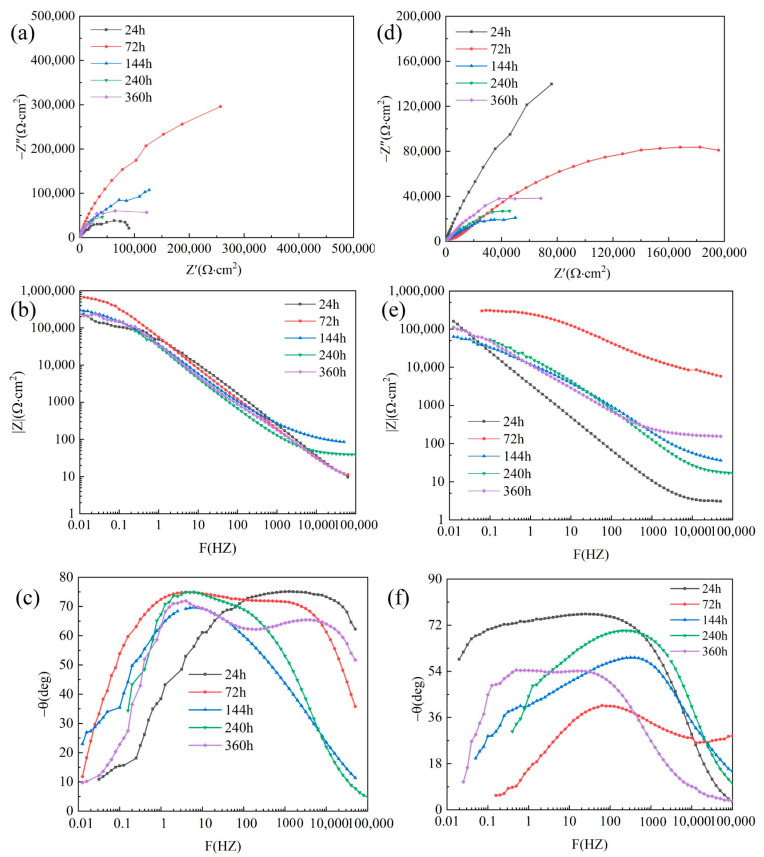
EIS of Al_2_O_3_3D/5083 Al(**d**–**f**) and EIS of 5083Al alloy (**a**–**c**) after NSS at different times: (**a**,**d**) Nyquist diagram; (**b**,**e**) Bode diagram (|Z|-F); (**c**,**f**) Bode diagram (−θ-F).

The EIS of corrosion products was conducted to study the influence of corrosion product layer on the material surface of the corrosion process. As shown in Figure 10a,d, the Nyquist diagram of 5083 Al, the high- and medium-frequency capacitance resistance arc after corrosion, the capacitance resistance in the high-frequency region was larger at 72 h, and it gradually decreased with time. The Al_2_O_3_3D/5083 Al Nyquist plot consisted of a semicircular inductive resistance arc. The high- and medium-frequency capacitive–resistance arcs corresponded to the corrosion products on the surface of Al_2_O_3_3D/5083 Al. The low-frequency capacitance–resistance arc corresponded to the electrochemical corrosion reaction on the electrode surface. With an increase in salt spray time, the capacitance resistance of Al_2_O_3_3D/5083 Al in the high-frequency area decreased rapidly from 24 h to 144 h, and corrosion products were slowly formed on the surface after 240 h. The capacitance resistance in the high-frequency area increased slowly. The impedance modulus of Al_2_O_3_3D/5083 Al exhibited a rise, then a fall, and then a rise again at high frequency. This pattern was the same as the phase angle, and corrosion resistance was enhanced at 240 h. 

### 3.6. Tafel of Corrosion Product

The Tafel of corrosion products was performed to study the effect of corrosion products on corrosion performance. Galvanic coupling corrosion is a key issue in the localized corrosion of Al alloys. It is caused by the nonhomogeneous microstructure of these alloys. Pitting corrosion caused by galvanic coupling reactions may be the initiation point of cracks, leading to failure. The current interactions depend on their electrochemical properties, which are closely related to the surrounding environment and are the key to understanding corrosion formation. The electro-couple interaction between the two phases can lead to crater formation through the dissolution of the particles or the corrosion of the substrate adjacent to the particles. 

**Figure 11:**
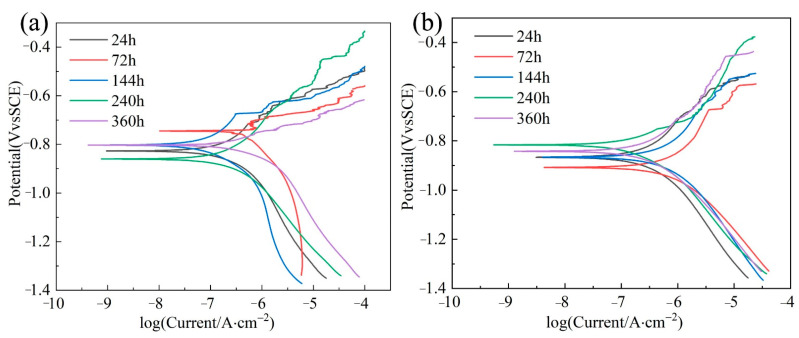
(**a**) Tafel of 5083Al after NSS at different times; (**b**) Tafel of Al_2_O_3_3D/5083Al after NSS at different times.

As shown in Figure 11a, the corrosion potential of 5083 Al exhibited a successive decline followed by a rise. The corrosion anode demonstrates evident fluctuations. The corrosion reaction was more obvious. 

At 72 h, the metal passivation phenomenon appeared, corrosion potential increased, corrosion tendency decreased, corrosion current density was reduced, and corrosion resistance improved. Corrosion resistance was maintained better. 

Figure 11b shows the corrosion potential of Al_2_O_3_3D/5083 Al at different times. Corrosion current density was larger, and then smaller, and then larger again. The phenomenon of anodic passivation appeared after 72 h, but the addition of Al_2_O_3_3D reduced the exposed area of the metal, weakening the passivation effect of the substrate Al. Corrosion potential decreased, and corrosion current density rose, with a decrease in corrosion resistance. 

Ten samples were tested using electrochemistry, and the results listed in Table 3 and Table 4. Corrosion current density of the 5083 Al after different times that NSS was 2.115 µA·cm^2^, 1.024 µA·cm^2^,1.086 µA·cm^2^, 2.114 µA·cm^2^, and 2.580 µA·cm^2^, respectively, for the 24 h, 72 h, 144 h, 240 h, and 360 h of NSS, as shown in Table 3. Corrosion current density of the Al_2_O_3_3D/5083 Al after different time NSS was 2.808 µA·cm^2^, 7.048 µA·cm^2^, 5.343 µA·cm^2^, 1.094 µA·cm^2^, and 2.823 µA·cm^2^, respectively, for the 24 h, 72 h, 144 h, 240 h, and 360 h of NSS, as shown in Table 4. 

Table 3 and Table 4 exhibited the phenomenon of passivation of 5083 Al metal at 72 h with enhanced corrosion resistance and weakened corrosion resistance of the composite material at 72 h. In addition, 5083 Al with weakened corrosion resistance at 240 h and a decrease in corrosion rate of Al_2_O_3_3D/5083 Al at 240 h with enhanced corrosion resistance. The residual salt crystal accumulated at 24–72 h of the Al_2_O_3_3D/5083 Al, Al matrix corrosion resistance was weakened. A large number of corrosion products were generated, and corrosion products had uneven distribution. Al atoms produced by corrosion were oxidized into Al (OH)_3_ with O_2_, which coated the Al matrix surface and hindered the corrosion reaction. After 240 h of the NSS experiment, the corrosion products of 5083 Al matrix in Al_2_O_3_3D/5083 Al are covered on the surface of Al_2_O_3_3D/5083 Al, which improves the corrosion resistance of the Al_2_O_3_3D/5083 Al. After the 144–240 h NSS experiment, the number of Mg_2_Si had increased. Mg_2_Si are one of the second phases in the Al matrix, which can be used as a corrosion anode. The increase of Mg2Si will enhance the corrosion resistance of Al_2_O_3_3D/5083 Al. After 360 h of the NSS experiment, corrosion current density increased and the corrosion resistance of Al_2_O_3_3D/5083 Al decreased. 

### 3.7. Analysis of Corrosion Rate (CR)

CR is calculated by weight loss in accordance with ASTM-G31-72:

CR = (K × W)/(A × T × D),(1)

where the constant K = 8.76 × 103, W is the weight loss, A is the area of the sample exposed to the NaCl solution, T is the exposure time, and D is the standard density of the material under test. In this experiment, the dimensions of the parallel hexahedral sample are 9 × 9 × 5 mm^3^. The exposed surface of the sample is 81 mm^2^. The density of Al_2_O_3_3D/5083 Al is 2.19 g/cm^3^, while the density of the 5083 Al alloy is 2.71 g/cm^3^.

**Figure 12:**
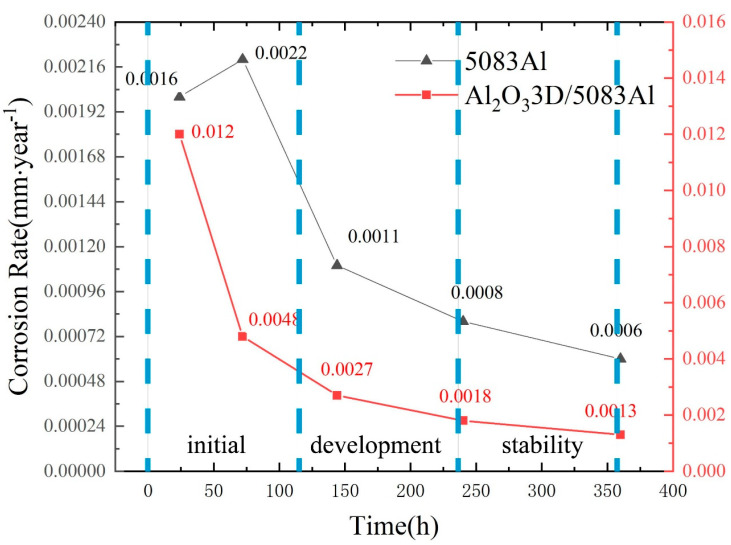
Corrosion rate (CR) of the two materials vs. NSS time.

The variation of CR with NSS time for both materials in Figure 12 shows that the weight loss of the 5083 Al alloy is less than that of the Al_2_O_3_3D/5083 Al composite. It illustrates the corrosion development process can be divided into the initial period, the development period, and the stability period. Compared with the 5083 Al alloy, (1) the skeleton of Al_2_O_3_3D is also capable of corrosion, and corrosion will lead to loose and porous bone; and (2) the skeleton structure of Al_2_O_3_3D is not sufficiently dense, pores occur, and after a long period of corrosion, the Al_2_O_3_3D skeleton appears to shed Al_2_O_3_ particles.

## 4. Discussion

The corrosion resistance of Al_2_O_3_3D/5083 Al was lower than that of 5083 Al alloy during the initial period, higher than that of 5083 Al alloy during the development period, and there is no obvious difference in corrosion resistance during the stability period. 

The Al interface prepared by the Al_2_O_3_3D/5083 Al composite exhibits poor corrosion resistance, uneven elemental division in the 5083 Al matrix, more residual stress in the Al_2_O_3_3D/5083 Al, and the existence of pores in the Al interface, leading to the decreased corrosion resistance of Al_2_O_3_3D/5083 Al [28].

Corrosion tendency is expressed by the corrosion potential. The higher the corrosion potential, the lower the corrosion tendency. Electrochemical test results show that the Al_2_O_3_3D/5083 Al composite exhibits higher corrosion tendency during the initial period, and the corrosion resistance of the material is better than that of the 5083 Al alloy. When the corrosion reaction is implemented, the Al_2_O_3_3D/5083 Al composite has poorer corrosion resistance than 5083 Al due to the Al matrix. During the corrosion development period, corrosion occurs within the Al_2_O_3_3D/5083 Al composite products, together with the newly generated Al (OH)_3_. Corrosion resistance is better than that of the 5083 Al alloy. During the corrosion stability period, the Al_2_O_3_3D skeleton combines with the newly generated Al (OH)_3_, and the material promotes excellent corrosion resistance. The corrosion resistance is close to that of the 5083 Al alloy. The research on the corrosion mechanism of the Al_2_O_3_3D/5083 Al alloy IPC under Cl^-^ conditions can explore the methods to protect the composite, and improve the service life of the composite under coastal and salt spray conditions. Therefore, the Al_2_O_3_3D/5083 Al alloy IPC can not only meet the mechanical and friction properties of the brake disc under different braking conditions, but also ensure its corrosion resistance, which is of great significance for the safe application of composites of brake discs [29].

## 5. Conclusions

1. The corrosion development process can be divided into the initial period, the development period, and the stability period.
2. The OCP, PDP curves, and EIS tests on the sample of polished surface show that the corrosion resistance of the Al_2_O_3_3D/5083 Al is better than that of 5083 Al.
3. The NSS test shows that the corrosion resistance of Al_2_O_3_3D/5083 Al was lower than that of the 5083 Al alloy during the initial period of corrosion and higher than that of the 5083 Al alloy during the corrosion development period.
4. Al_2_O_3_3D used as a reinforcement in the Al_2_O_3_3D/5083 Al composite improves the corrosion resistance of the Al_2_O_3_3D/5083 Al composite. The interpenetrating structures of Al_2_O_3_3D and the 5083 Al matrix, combined with the strong interface, are not easy to corrode. Al_2_O_3_3D and the 5083 Al matrix are combined tightly to promote excellent corrosion resistance.

## Figures and Tables

**Table 1:** Composition of 5083 Al alloy (mass fraction).

Elements	Si	Cu	Mg	Zn	Mn	Ti	Cr	Fe	Al
Wt.%	0.14	0.03	3.90	0.02	0.60	0.15	0.07	0.42	Balance

**Table 2:** Corrosion potential and corrosion current.

Sample	Open Circuit Potential OPC (mV)	E_corr_ (mV)	I_corr_ (µA·cm^2^)
Al_2_O_3_3D/5083Al	−791.6	−1069	6.410
5083Al	−773.9	−849	9.879

**Table 3:** Tafel of 5083 Al after NSS at different times.

Tafel of 5083 Al after NSS at Different Times					
Time/h	24	72	144	240	360
Ecorr/mV	−827	−720	−803	−860	−803
Icorr/(µA·cm^2^)	2.115	1.024	1.086	2.114	2.580

**Table 4:** Tafel of Al_2_O_3_3D/5083 Al after NSS at different time.

Tafel of Al_2_O_3_3D/5083 Al after NSS at Different Times					
Time/h	24	72	144	240	360
Ecorr/mV	−867	−908	−866	−816	−842
Icorr/(µA·cm^2^)	2.808	7.048	5.343	1.094	2.823

## Data Availability

Data sharing is not applicable for this article.
